# Chlamydia protein Pgp3 studied at high resolution in a new crystal form

**DOI:** 10.1107/S2052252518007637

**Published:** 2018-06-07

**Authors:** Sahir Khurshid, Lata Govada, Gillian Wills, Myra O. McClure, John R. Helliwell, Naomi E. Chayen

**Affiliations:** a Computational and Systems Medicine, Department of Surgery and Cancer, Imperial College London, Sir Alexander Fleming Building, South Kensington, London SW7 2AZ, England; b Department of Medicine, Imperial College London, St Mary’s Campus, London W2 1PG, England; c School of Chemistry, The University of Manchester, Manchester M13 9PL, England

**Keywords:** chlamydia protein, Pgp3, crystallization, crystal form, protein structure, X-ray crystallography, structural biology, sexually transmitted diseases

## Abstract

The protein Pgp3 is implicated in the sexually transmitted disease chlamydia and comprises an extended complex arrangement of a C-terminal domain (CTD) and an N-terminal domain (NTD) linked by a triple-helix coiled coil (THCC). Here, the X-ray crystal structure of Pgp3 from an LGV1 strain is reported at the highest X-ray diffraction resolution obtained to date for the full protein. The protein was crystallized using a high concentration of potassium bromide, which resulted in a new crystal form with relatively low solvent content that diffracted to a resolution of 1.98 Å. The three-dimensional structure of this new crystal form is described and compared with those of other crystal forms, and the potassium bromide binding sites and the relevance to chlamydia isolates from around the globe are described. The crystal packing is apparently driven by the CTDs. Since the threefold axes of the THCC and NTD are not collinear with the threefold axis of a CTD, this naturally leads to disorder in the THCC and the portion of the NTD that does not directly interact with the CTD *via* crystal packing. The key avenue to resolving these oddities in the crystal structure analysis was a complete new analysis in space group *P*1 and determining the space group as *P*2_1_2_1_2_1_. This space-group assignment was that originally determined from the diffraction pattern but was perhaps complicated by translational noncrystallographic symmetry. This crystal structure of a three-domain multi-macromolecular complex with two misaligned threefold axes was a unique challenge and has not been encountered before. It is suggested that a specific intermolecular interaction, possibly of functional significance in receptor binding in chlamydia, might allow the design of a new chemotherapeutic agent against chlamydia.

## Abstract 2

Pgp3, a protein implicated in the sexually transmitted disease chlamydia, is described in a new crystal structure. It comprises a three-domain multi-macromolecular complex with two misaligned threefold axes; this comprised a unique challenge that has not been encountered before. A specific intermolecular interaction, possibly of functional significance in receptor binding in chlamydia, might allow the design of a new chemotherapeutic agent against chlamydia.

## 1. Introduction   

*Chlamydia trachomatis* (Ct) is a common bacterium infecting animals and humans, with infection being a leading cause of ocular and urogenital disease. Recent serological studies have revealed that Ct infection is often asymptomatic and/or undiagnosed (Horner *et al.*, 2016 ▸). This is of concern as the disease can lead to serious sequelae if left untreated, particularly in women. Pelvic inflammatory disease can develop, leading to tubal factor infertility and ectopic pregnancy (Haggerty *et al.*, 2010 ▸; Price *et al.*, 2013 ▸).

Nearly all clinical isolates of Ct contain several copies of a highly conserved plasmid (Thomas *et al.*, 1997 ▸). This plasmid has been associated with the pathogenicity of the organism; in both mice and primates, infection with plasmid-free strains results in less severe pathology both in the eye and the upper genital tract (Carlson *et al.*, 2008 ▸; Kari *et al.*, 2011 ▸; O’Connell *et al.*, 2011 ▸; Olivares-Zavaleta *et al.*, 2010 ▸; Sigar *et al.*, 2014 ▸). The plasmid is not required for pathogenicity by all species of *Chlamydia*, however, and is rarely found in *C. pneumoniae* isolates (Pickett *et al.*, 2005 ▸). There is no association between the presence of the plasmid in *C. caviae* and virulence in guinea pigs (Frazer *et al.*, 2012 ▸). When investigated for their potential as attenuated vaccines, plasmid-less Ct strains conferred partial protection against subsequent infection, but some animals remained vulnerable. Further investigation is needed to discover why this was not replicated in genital strains (Patton *et al.*, 2015 ▸), although plasmid-less ocular strains stimulate a protective immune response in some primates (Kari *et al.*, 2011 ▸). This incomplete protection could be partly explained by the finding that plasmid-free strains could not stimulate Toll-like receptor 2 (TLR-2)-dependent immune responses in mice (O’Connell *et al.*, 2011 ▸).

The Ct plasmid encodes eight proteins, three of which are unique to *Chlamydia* and have unknown function. One of these, the Pgp3 protein, is transcribed from the CDS5 gene. This protein is both associated with the bacterial outer membrane and secreted into the cell cytosol (Li, Chen *et al.*, 2008 ▸), and is thought to be a virulence and *in vivo* fitness factor supporting Ct infection. It is involved in stimulating host immune responses. In a mouse model, reintroduction of the intact plasmid into a plasmid-less strain restored infectivity and inflammatory responses. However, when the plasmid-free Ct strain was transformed with a CDS5-knockout plasmid, infectivity and stimulation of an immune response was not re-established (Ramsey *et al.*, 2014 ▸).

X-ray crystallographic studies of Pgp3 from a genotype D strain of Ct have been reported at a resolution limit of 3.1 Å by Galaleldeen *et al.* (2013 ▸), establishing that the 84 kDa Pgp3 trimer is comprised of globular N- and C-terminal domains connected by a triple-helical coiled coil (THCC). The authors suggested that although the Pgp3 protein has no definite homologues, its C-terminal structure shows similarities to the tumour necrosis factor family of cytokines and contains ‘hotspots’ for protein–protein interactions. These are likely to be responsible for the distinct immunogenicity of the protein in its native, trimeric form (Bas *et al.*, 2001 ▸; Chen, Lei *et al.*, 2010 ▸; Comanducci *et al.*, 1994 ▸; Li, Zhong *et al.*, 2008 ▸). In addition, Galaleldeen *et al.* (2013 ▸) suggested that the lectin-like N-terminal domain of Pgp3 could adhere to the chla­mydial cell to aid host cell invasion. Recently, Hou *et al.* (2015 ▸) reported that Pgp3, specifically its central region, neutralizes the activity of cathelicidin LL-37, an antichlamydial peptide secreted by epithelial cells and neutrophils in the human genital tract. The Pgp3 secreted into the host-cell cytosol is likely to be released when infected cells lyse to counteract antimicrobial host effects; this is consistent with the Pgp3 protein being highly immunogenic.

Another interesting aspect involves its serovars, a term that is used to allow organisms to be classified at the subspecies level; this aspect includes an issue of particular importance in epidemiology. There is a co-evolutionary relationship between the host organism serovar and its plasmid (Seth-Smith *et al.*, 2009 ▸), indicating that the plasmid has a preferential tropism for its own host (Song *et al.*, 2014 ▸). However, this preference was less obvious in another study (Ramsey *et al.*, 2014 ▸), and plasmids from different serovars appear to be functionally interchangeable between Ct isolates (Ramsey *et al.*, 2014 ▸). Given the need for further understanding of the different Ct serovars and their plasmids, and the increasing evidence that Pgp3 is an important virulence factor, evaluation of the similarities and differences of the protein between serovars is required, together with more detailed analysis of the structure than is currently available. The Chlamydia Biobank (http://www.chlamydiabiobank.co.uk/Home.html) is actively gathering new isolates from around the world.

We report the X-ray crystal structure analysis of Pgp3 from an LGV1 strain at the highest X-ray diffraction resolution obtained to date for the full protein.

## 2. Methods   

### 2.1. Protein expression   

A DNA plasmid construct was produced for the expression of Pgp3 from an LGV1 strain protein (Pgp3L1) with an N-terminal GST tag. A DNA fragment encoding Pgp3L1 was PCR-amplified from pCTL1 2A, a construct encoding the entire LGV1 (strain 440) *C. trachomatis* plasmid (Hatt *et al.*, 1988 ▸), using *Pfu* DNA polymerase (Stratagene, USA) and the primer pairs 5′-CGTAGGATCCATGGGAAATTCTGGTTT-3′ and 5′-CGTACTCGAGTTAAGCGTTTGTTTGAGGT-3′. The PCR amplicon was digested with BamHI and XhoI and ligated into the multiple-cloning site of pGEX-4T-1 (Stratagene) to produce the construct pGEX-Pgp3L1.

The Pgp3-GST fusion protein was then expressed in an *Escherichia coli*-based system. The endonuclease A-deficient *E. coli* strain PC2 [BL21 (DE3), *endA*::Tet^R^, T1^R^, pLysS] (Cherepanov, 2007 ▸) was transformed with the pGEX-Pgp3L1 construct and expression of the GST-Pgp3L1 fusion protein was induced overnight at 18°C with 0.25 m*M* isopropyl β-d-1-thiogalactopyranoside (Sigma–Aldrich, UK).

Pgp3 is a monomer of 28 kDa and is known to form trimers of 792 residues. The accession number for Pgp3L1 is YP_001569038.

### 2.2. Purification   

Bacterial lysates containing the GST-Pgp3L1 fusion protein were disrupted by sonication in buffer *A* (200 m*M* NaCl, 50 m*M* Tris–HCl pH 7.4, 0.5 m*M* EDTA) containing 0.5 m*M* phenylmethylsulfonyl fluoride and 5 m*M* dithiothreitol (DTT). Crude extracts pre-cleared by centrifugation were incubated with glutathione Sepharose (GE Healthcare Life Sciences, UK) and the resin was extensively washed in buffer *A*. The GST tag was cleaved from the protein using thrombin (3 U per milligram of protein) for 4 h at 25°C and the protein was eluted with buffer *A*. Protein-containing fractions diluted with three volumes of 50 m*M* Tris–HCl pH 7.4 were injected into a 5 ml HiTrap Q FF column (GE Healthcare) on an ÄKTApurifier (GE Healthcare). The bound protein was eluted with a linear gradient of 0.15–0.3 *M* NaCl in 50 m*M* Tris–HCl pH 7.4, and 5 m*M* DTT was then added to each 1 ml fraction. The pooled protein-rich fractions were further concentrated twofold to fivefold by ultrafiltration in a Centriprep Ultracel YM-3 (3000 molecular-weight cutoff) column (Millipore, UK). Protein concentration was determined by the Bradford assay with bovine serum albumin as a standard (Bio-Rad, UK) and aliquots (2 µl) were denatured in 2× SDS buffer (Laemmli, 1970 ▸) for 5 min at 95°C and analysed by PAGE on a 10% gel.

### 2.3. Crystallization   

A 7 mg ml^−1^ Pgp3 stock buffered with 220 m*M* NaCl, 50 m*M* Tris–HCl pH 7.5, 5 m*M* DTT was screened for suitable crystallization conditions in sitting-drop vapour-diffusion format using a Mosquito robot (TTP Labtech, UK). A variety of commercially available screens such as Crystal Screen HT, Index HT (Hampton Research, USA), Morpheus and PGA (Molecular Dimensions, UK) were used in 96-well MRC crystallization plates (Molecular Dimensions, UK). All screening trials contained 400 nl drops, each comprised of a 1:1 ratio of the protein and screen solutions. The crystallization trials were incubated at 293 K.

The different leads obtained from the crystallization screening trials were optimized by determining working phase diagrams as detailed in Saridakis & Chayen (2000 ▸). Each optimization trial was also scaled up to 1 µl drop volume and set up manually in hanging drops (Qiagen NeXtal plates) and in microbatch experiments (Chayen, 1999 ▸).

A two-step cryoprotection of the crystals was performed by soaking them in a solution consisting of the crystallization condition and 10% glycerol for 10 s and transferring them to the crystallization condition containing 20% glycerol for a further 10 s. The cryoprotected crystals were then vitrified in liquid nitrogen in preparation for diffraction analysis. All crystals were initially tested in-house on a Rigaku MicroMax-007 HF M high-flux Cu *K*α generator coupled with a Rigaku Saturn 944+ CCD detector. Crystals were grown again under the optimal conditions and transported to Diamond Light Source from X-ray data collection.

### 2.4. Data collection and processing   

A complete X-ray diffraction data set was collected on beamline I04 at Diamond Light Source using a Dectris PILATUS 6M-F detector (Table 1 ▸) with the wavelength (0.9173 Å) chosen to optimize the bromine anomalous differences by utilizing the Br *K* edge. 1800 X-ray diffraction data images each of 0.2° rotation, forming a full angular revolution of the crystal, were processed with *MOSFLM* and the data were scaled in *SCALA* from the *CCP*4 suite (Winn *et al.*, 2011 ▸). The raw diffraction images have been deposited at Zenodo (https://doi.org/10.5281/zenodo.1248459).

### 2.5. Crystal structure determination   

Excellent molecular-replacement-based structures were obtained using *Phaser* (McCoy *et al.*, 2007 ▸) with both the CTD and the full Pgp3 from PDB entry 4jdm (Galaleldeen *et al.*, 2013 ▸). In each case the *Phaser* molecular-replacement statistics gave clear solutions in space group *P*2_1_2_1_2_1_, as already firmly indicated from the X-ray diffraction Bragg reflection-intensity systematic absence conditions in the *POINTLESS*/*SCALA* diffraction data merging.

The two Pgp3 copies are comprised of polypeptide chains *A*, *B*, *C* and *D*, *E*, *F*. The composite OMIT electron-density map showed excellent coverage of the CTD for both Pgp3 copies. This guided our approach to the full molecular structures. The NTD was clear for chains *D*, *E* and *F*, whereas for chains *A*, *B* and *C*, although evident in terms of 2*F*_o_ − *F*_c_ electron density, the NTD showed a small but significant shift in position based on the *F*_o_ − *F*_c_ electron-density map, which we first tried to remodel and then to simply shift as a rigid-body refinement in *REFMAC*5 (Murshudov *et al.*, 2011 ▸). We also tried rerunning *Phaser* with the *A*, *B*, *C* subunits separated into the NTD and THCC. This did not yield an improved solution.

A composite OMIT map was also calculated using the CTD of PDB entry 4jdn (Galaleldeen *et al.*, 2013 ▸) and clearly showed electron density for the NTD in the correct position for lattice interactions with a CTD. The presence of the THCC in both copies was unclear in terms of electron-density evidence and the atoms had very high *B* factors; it was inspected residue by residue. There was more evidence for the presence of the THCC in chains *D*, *E* and *F* than in chains *A*, *B* and *C*, *i.e.* there was some, albeit quite broken, electron density along portions of the THCC polypeptide in chains *D*, *E* and *F*.

A detailed protein model refinement of the full Pgp3 molecular-replacement solution was then performed using *REFMAC*5 (Winn *et al.* 2011 ▸); see Table 1 ▸. Protein model validation was undertaken using *MolProbity* (Chen, Arendall *et al.*, 2010 ▸), with added H atoms in stereochemically predictable positions and some amino-acid side chains ‘flipped’ for likely hydrogen bonds, as well as using *Coot* (Emsley *et al.*, 2010 ▸). Subsequently, the PDB validation report emphasized that the lack of visibility of the THCC led to a poor-quality geometric definition of these amino acids. The portions of the NTD attached to the THCC were likewise affected. The portions of the NTD that interact with the CTD in their crystal packing, however, were well determined. Basically, as one moves away from the crystal contact interface of the CTD and the ordered portion of the NTD, the quality of fit of the polypeptide deteriorates. We have removed the clearly disordered portions of the model, but this is a progressive effect as the distance from the interface increases. It is obvious that the CTD in each Pgp3 molecule is well ordered and this further supports the idea that it is the CTD that is controlling crystallization under our high-salt conditions. As a further check we expanded the diffraction data into *P*1 using *CCP*4 and then reran the *Phaser* MR in *P*1. This was important because it freed the *Phaser* MR calculation from any constraint of the *P*2_1_2_1_2_1_ calculation. Using *Zanuda* in *CCP*4 (Lebedev & Isupov, 2014 ▸), the solution obtained allowed direct confirmation that the correct space group was *P*2_1_2_1_2_1_.

We have deposited the 1.98 Å resolution crystal structure of the ordered parts in the PDB. For completeness, the full molecular structure to the full diffraction resolution of 1.98 Å is summarized in Table 1 ▸. These coordinates and structure factors are also provided as Supporting Information. We believe that the reason for the loss of order in a THCC and the NTD connected to it is owing to the crystal packing being driven by the CTDs. Since the threefold axes of the THCC and NTD are not collinear with the threefold axis of a CTD, this naturally leads to disorder in the THCC and the portion of the NTD that is not directly interacting with the CTD *via* crystal packing. The PDB coordinate file is therefore the ordered part of the crystallographic structure. The Supporting Information includes this ordered structure accompanied by what we envisage to be the most likely full model.

## 3. Results   

**Figure 1:**
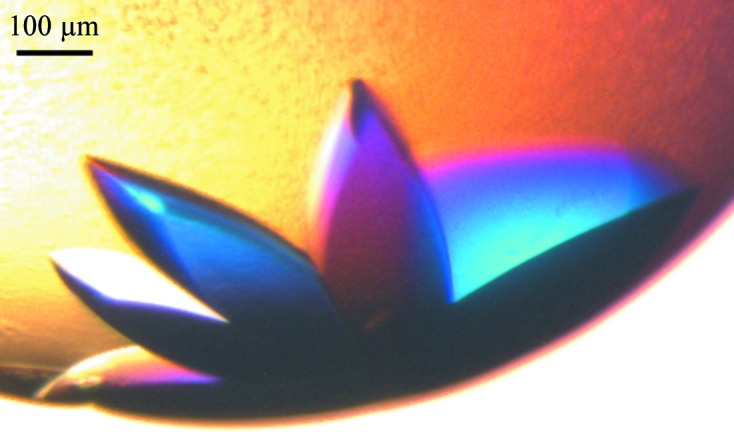
Typical Pgp3 crystals.

**Figure 2:**
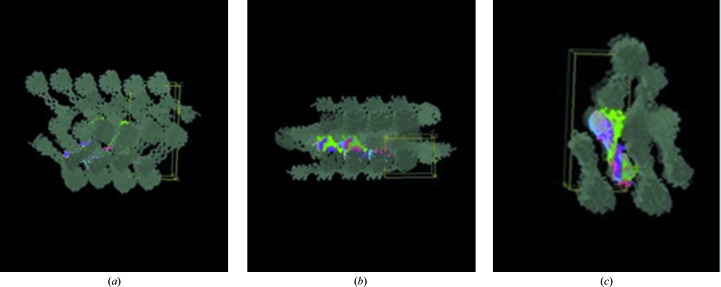
Crystal-packing diagrams of Pgp3 in three orthogonal directions. This figure was prepared using *Coot* (Emsley *et al.*, 2010 ▸). (*a*) View down **a** with the **c** axis vertical and the **b** axis horizontal. (*b*) View down **c** with the **a** axis vertical and the **b** axis horizontal. (*c*) View down **b** with the **c** axis vertical and the **a** axis horizontal.

Typical crystals of Pgp3 are shown in Fig. 1 ▸. This new crystal form belongs to space group *P*2_1_2_1_2_1_. The crystal packing of the two full Pgp3 molecules is shown in Fig. 2 ▸ viewed along the three crystallographic dimensions. This shows good crystal packing and also the translational noncrystallographic symmetry. The full-length Pgp3 in this *P*2_1_2_1_2_1_ crystal form is compared with that in PDB entry 4jdm (Galaleldeen *et al.*, 2013 ▸). The structures belonged to different space groups with different unit-cell dimensions. The solvent content for PDB entry 4jdm was 73.8%, whereas our new crystal form is more tightly packed with a solvent content of 54%, but both have two molecules in the crystal asymmetric unit. These crystal solvent contents, with that for PDB entry 4jdm being larger and that reported here being smaller, are very likely to account for the respective X-ray diffraction resolution limits of 3.1 Å *versus* 1.98 Å.

The LGV1 isolate shows amino-acid sequence changes from the genital isolate as seen in the electron-density map (see Supplementary Fig. S1). The amino-acid changes are (with the LGV1 amino-acid one-letter code given first and the genital isolate amino-acid one-letter code given second) Q12E, T39K, P61S, D86N, Q90D, R138S, Y191C, R210S and I212T. The X-ray electron-density maps (2*F*_o_ − *F*_c_ and *F*_o_ − *F*_c_ based on the sequence of the genital isolate; PDB entry 4jdm) clearly showed that these changes were needed in each of the ordered portions of the *A*, *B*, *C*, *D*, *E* and *F* polypeptide chains, namely R138S, Y191C, R210S and I212T. There was evidence for Q12E, D86N and Q90D changes in some of the polypeptide chains but not all, and the T39K and P61S substitutions were not visible owing to their high atomic displacement (*B*) factors. All amino-acid changes were made in any case to the atomic coordinates for all chains based on the LGV1 amino-acid sequence.

### 3.1. Cation-binding sites   

**Figure 3:**
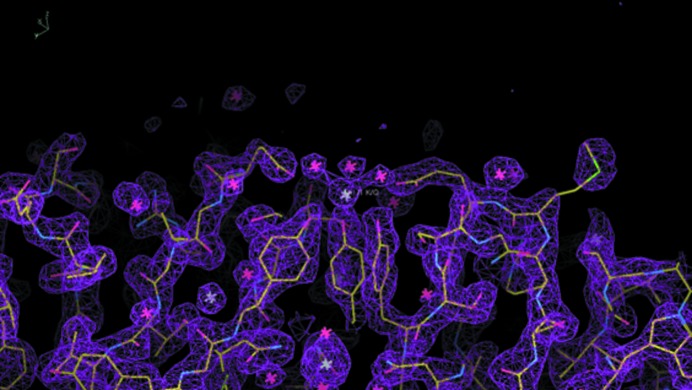
The K^+^ cation-binding site at Tyr197 in molecule 1.

The predominant cation-binding site is a potassium ion in an octahedral coordination involving each O atom of the three tyrosine side chains Tyr197 and three bound water-molecule O atoms (Fig. 3 ▸). A previous study also reported this as a potassium-binding site (Galaleldeen *et al.*, 2013 ▸), and this alkali ion-binding site was also reported in PDB entry 4jdo (in each of the three molecules in the crystallographic unit), in this case containing sodium.

According to the electron-density map evidence, and the local structural environment of each peak, a total of six potassium-binding sites have been identified in our study, including the one referred to above.

### 3.2. Anion-binding sites   

**Figure 4:**
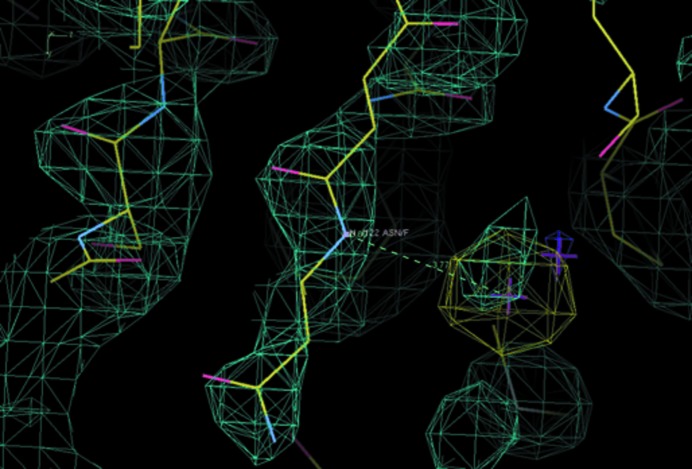
An example of an anion-binding site: Asn122 in protein subunit *F*. The anomalous difference Fourier map contoured at 4σ is shown in yellow and the composite OMIT electron-density map contoured at 1.6 r.m.s. is shown in turquoise; both neatly show the bromide anion. This figure was prepared using *Coot* (Emsley *et al.*, 2010 ▸).

According to the electron-density map evidence, 2*F*_o_ − *F*_c_ and anomalous difference electron density derived from diffraction data measured on the high-energy side of the Br *K*-edge peak to enhance the bromine signal, as well as the local structural environment of each peak, a total of 28 bromide ion-binding sites have been identified in our study. We illustrate these with an example of a hydrogen bond to a peptide NH at Asn122 as shown in Fig. 4 ▸. We note that the ordered bromine sites are in the ordered protein portions and not elsewhere. We tried several different crystals and each showed the same distribution of the anions linked to the polypeptide disorder discussed above.

### 3.3. Crystal lattice interactions   

#### 3.3.1. Phe6–Trp234 interaction   

**Figure 5:**
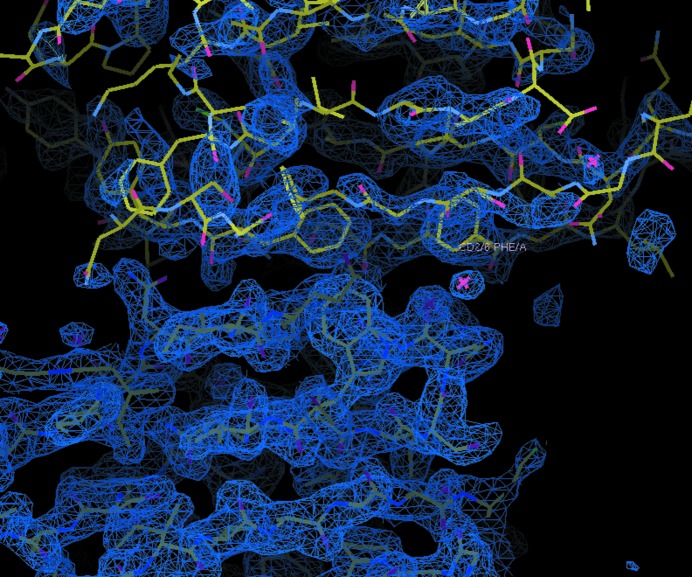
The close interaction of Trp234 in the CTD of subunit *D* with a symmetry-related Phe6 in the NTD of subunits *D*, *E* and *F*. This figure was prepared using *Coot* (Emsley *et al.*, 2010 ▸).

The close interaction of these two amino acids seen in PDB entry 4jdm is also seen in our crystal form; see Fig. 5 ▸ for the interaction of Trp234 in the CTD of subunit *D* with Phe6 in a symmetry-related NTD of subunits *D*, *E* and *F* (and likewise for the interaction of Trp234 in the CTD of subunit *A* with Phe6 in a symmetry-related NTD of subunits *A*, *B* and *C*).

#### 3.3.2. The translational noncrystallographic symmetry (TNCS)   

**Figure 6:**
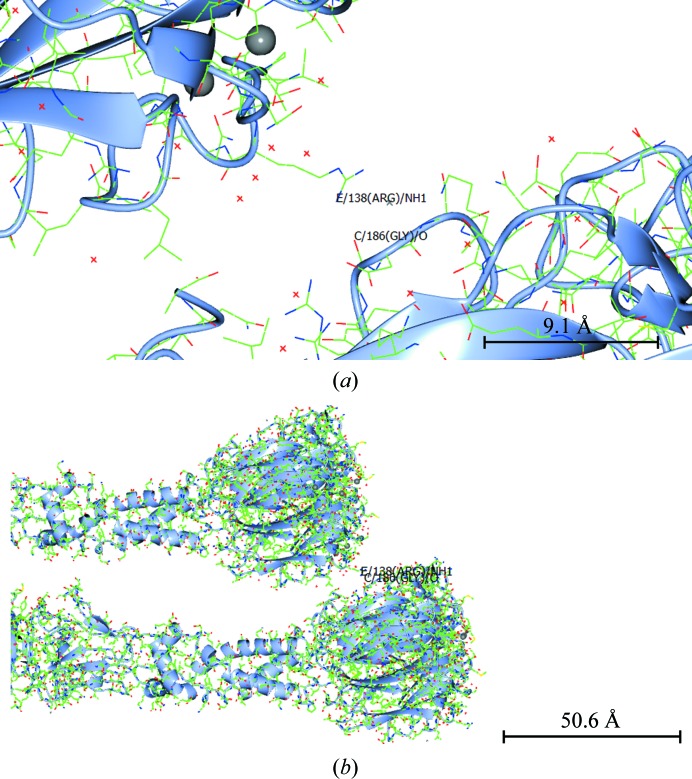
The translational noncrystallographic symmetry (TNCS). The TNCS is mediated by a single hydrogen bond between Arg138 NH and the carbonyl of Gly186 and has a hydrogen-bond distance of 2.7 Å. (*a*) Close-up view, (*b*) zoomed-out view. This figure was prepared with *CCP*4*mg* (McNicholas *et al.*, 2011 ▸). The standard uncertainty in this hydrogen-bond distance is 0.5 Å and was calculated using *Online_DPI* (Kumar *et al.*, 2015 ▸).

The TNCS is mediated by a single hydrogen bond between Arg138 NH and the carbonyl of Gly186 (Fig. 6 ▸); this hydrogen bond has a distance of 2.7 Å from Arg138 in the *E* chain to Gly186 in the *C* chain.

#### 3.3.3. The packing interactions for the THCC   

**Figure 7:**
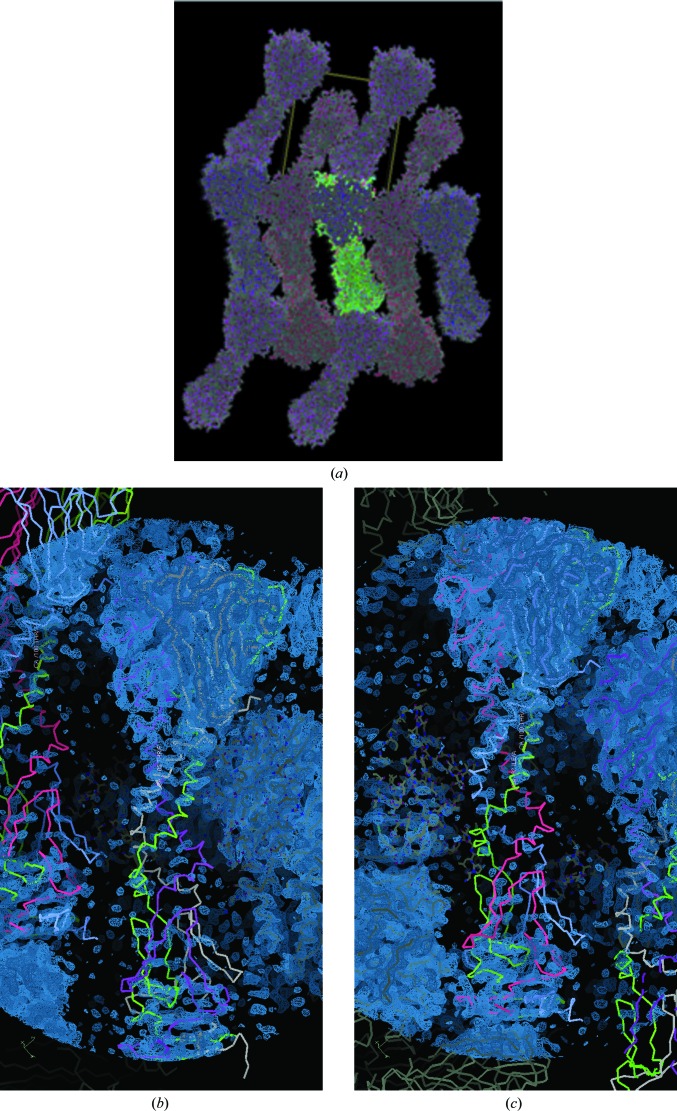
An analysis of the crystal-packing interactions. It is shown that the CTD and NTD are involved in packing in both molecules, but interactions are only made on one side for the THCCs. (*a*) Overall view, (*b*) enlargement for chains *D*, *E* and *F* and (*c*) enlargement for chains *A*, *B* and *C*. In (*b*) and (*c*) the composite OMIT map (2*F*_o_ − *F*_c_) contoured at 1.2 r.m.s. is shown in blue as calculated in *PHENIX* (Afonine *et al.*, 2012 ▸); the THHC in each of these clearly has little to no OMIT electron-density map coverage. The colour coding is used to distinguish each of the polypeptide chains. These figures were prepared using *Coot* (Emsley *et al.*, 2010 ▸).

An analysis of the crystal-packing interactions clearly shows that the CTD and NTD are involved in these in both molecules but that the THCC is only involved on one side (Fig. 7 ▸). The enlarged views in Figs. 7 ▸(*b*) and 7 ▸(*c*) show how the THCCs only make interactions on one side. These results confirm the possibility of flexibility of the linker between the CTD and NTD domains.

#### 3.3.4. The threefold Pgp3 noncrystallographic symmetry axis   

**Figure 8:**
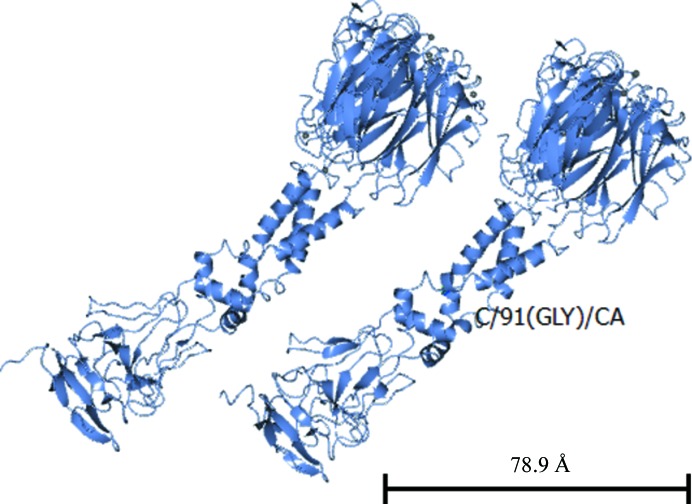
The threefold Pgp3 noncrystallographic symmetry axis. The view shows the angle between the NCS threefold axis for the CTD and the ‘top portion’ of the THCC inclined to the axis through the NTD and the ‘bottom portion’ of the THCC, which is approximately 15°; their intersection is approximately at amino-acid residue 91. This figure was prepared with *CCP*4*mg* (McNicholas *et al.*, 2011 ▸).

Fig. 8 ▸ shows the threefold Pgp3 noncrystallographic symmetry axis. The angle between the NCS threefold axis for the CTD and the ‘top portion’ of the THCC inclined to the axis through the NTD and the ‘bottom portion’ of the THCC is approximately 15°. Their intersection is approximately at residue 91.

## 4. Discussion   

### 4.1. Overall crystal structure and comparisons with previous crystal structures of Pgp3 and portions of Pgp3   

Our *P*2_1_2_1_2_1_ crystal form and X-ray diffraction data have a higher diffraction resolution of 1.98 Å for the full Pgp3 protein compared with PDB entry 4jdm. The lower solvent content of this new crystal form of 54%, *versus* 73.8% for PDB entry 4jdm, is a reasonable explanation for this improved diffraction. The lower solvent content here also results in an increased number of lattice interactions. Of special importance is the lattice interaction observed in both crystals that involves the three Phe6 residues on the fairly flat NTD surface and a single Trp234 on the curved surface of the CTD of the crystal lattice neighbour. Obviously in this case, where there are more lattice interactions overall, its importance is less in organizing the crystal. Furthermore, the crystal structure of the CTD + NTD fusion protein (PDB entry 4jdo) also shows the Phe6–Trp234 intermolecular interaction for each of the three molecules in the asymmetric unit of this crystal (one of these is shown in Supplementary Fig. S2).

Galaleldeen *et al.* (2013 ▸) have shown that there is flexibility of the THCC from a leverage site at Gly85. Our electron-density map of the THCC fades around Ala93, rather than Gly85. The electron-density map starts to order again around Met47. The threefold axis through the CTD and top portion of the THCC and the threefold axis through the NTD and the bottom portion of the THCC seem to intersect at approximately residue 91. Thus, these two aspects, the fade of the electron-density map and these threefold axes, from this new study compared with the proposal of Galaleldeen and coworkers for Gly85 as the flexibility hinge, suggest flexibility in where the hinge occurs. A suite of electron-density images of the three domains in the two independent molecules are shown in Supplementary Figs. S3–S6.

**Figure 9:**
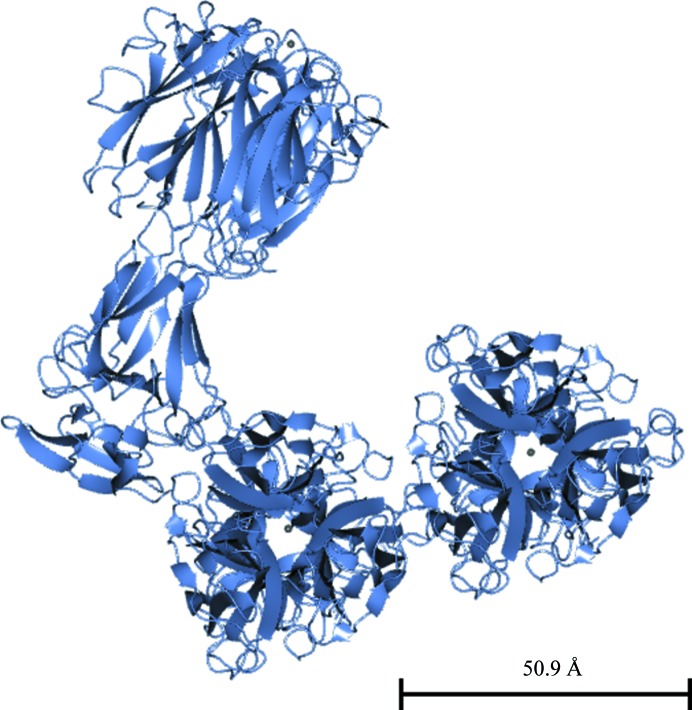
The crystal structure (PDB entry 4jdo) of the CTD + NTD fusion protein. The structure shows that the threefold axes of the CTD and the NTD are collinear; there are three molecules in the asymmetric unit in this crystal. Shown here are two of these viewed along their threefold axis (middle and right) and one perpendicular to it (extreme left). This figure was prepared with *CCP*4*mg* (McNicholas *et al.*, 2011 ▸).

For the full Pgp3 the threefold axis in the CTD and the ‘top portion’ of the THCC is inclined at an angle to that through the NTD. By contrast, in PDB entry 4jdo, the so-called fusion protein of the CTD and NTD, *i.e.* with the THCC removed, naturally shows a collinear threefold axis for both the CTD and the NTD (Fig. 9 ▸).

Insight into the reason for the presence of the disordered portions was provided by imagining of the crystallization of this molecule. Let us imagine the first Pgp3 molecule with the three CTD subunits labelled *A*, *B* and *C*. The next Pgp3 molecule with its CTD then comes along. Again, we label the CTD subunits *A*, *B* and *C*. The lattice interaction can form from *A* to *A*, from *A* to *B* or from *A* to *C*. At the other end of a Pgp3 molecule, the NTD then wants to attach to a CTD *via* the sticky patch. The fact that the threefold axes are not collinear leads to a disorder in the linking of the THCC and the NTD remote from the interface of the NTD and a CTD.

### 4.2. Salt-binding sites   

#### 4.2.1. Cation-binding sites   

We clearly see the presence of a potassium ion in an octahedral coordination involving each O atom of the three tyrosine side chains Tyr197 and three bound water-molecule O atoms (Fig. 3 ▸). This arrangement was first reported by Galaleldeen *et al.* (2013 ▸). This arose, chemically speaking, owing to the crystallization conditions used by Galaleldeen *et al.* (2013 ▸) for the CTD, which included sodium potassium phosphate at 1.2 *M* (PDB entry 4jdn). In the fusion protein comprising the CTD and the NTD the three molecules in the crystallographic asymmetric unit each have a sodium ion at this position. The octahedral coordination of the sodium ions reported in PDB entry 4jdo and the cation-to-ligand atom distances were a reasonable match to those expected. No other cation-binding sites (or anions) were reported.

#### 4.2.2. Anion-binding sites   

Individual examples of the Pgp3 bromide ion-binding sites, determined utilizing optimized anomalous differences at the Br *K* edge, have been described above. A total of 28 such ions were identified. These are basically all in the CTD. The most common form of interaction is *via* a hydrogen bond to a peptide NH. This has been highlighted before by Panjikar & Tucker (2002 ▸); see their porcine pancreatic elastase protein structure and bromide ions in PDB entry 1l0z.

A feature of the *B* factors for populations of both cations and anions is that they have values that are significantly higher than the bound waters (with *B* factors of 70, 64 and 32 Å^2^, respectively). This is presumably owing to the generally longer binding distance than a typical bound water hydrogen-bond distance, and naturally leads to higher *B* factors.

#### 4.2.3. Different isolates   

Our LGV1 isolate Pgp3 crystal structure shows where amino-acid changes actually occur (Q12E, T39K, P61S, D86N, Q90D, R138S, Y191C, R210S and I212T) compared with the Pgp3 crystal structure reported by Galaleldeen *et al.* (2013 ▸). Thus, we can say that Q12E is not near the structural cluster of three Phe6 residues and thus presumably is not functionally important. R210S and I212T are in the general vicinity of a Pgp3–Pgp3 intermolecular lattice contact in our crystal structure but again are not obviously functionally important. We conclude that The Chlamydia Biobank (http://www.chlamydiabiobank.co.uk/Home.html) makes a clear distinction between entries, and these two different Pgp3 crystal structures have therefore allowed a start on deciphering correlations between amino-acid sequence changes and the three-dimensional structure of Pgp3. These can now also be followed up actively as new isolates are collected and stored in The Chlamydia Biobank. Indeed, quoting from The Chlamydia Biobank,

diverse *C. trachomatis* isolates will be collected from researchers around the world.

Furthermore,

*C. trachomatis* isolates fall broadly into two biovars, namely LGV (lymphogranuloma venereum, MOMP serovars L1–L3) and trachoma (ocular strains, serovars A–C, and urogenital strains, serovars D–K). It is our intention that genome and plasmid sequences will be available for every living isolate stored in the Biobank.

Thus, these different isolates will have known gene sequences, including those of their Pgp3 proteins, and the positions of such amino-acid changes can now be readily mapped on the three-dimensional structure of Pgp3.

## 5. Conclusions   

A new crystal form of Pgp3 that diffracts to the highest resolution thus far observed has been obtained and described. The crystal packing is apparently driven by the CTDs. Since the threefold axes of the THCC and NTD are not collinear with the threefold axis of a CTD, this naturally leads to disorder in the THCC and the portion of the NTD that does not directly interact with the CTD *via* crystal packing. The key avenue to resolving these oddities in the crystal structure analysis was a complete new analysis in space group *P*1 and determination of the space group as *P*2_1_2_1_2_1_ using *Phaser* MR (McCoy *et al.*, 2007 ▸) and *Zanuda* (Lebedev & Isupov, 2014 ▸). This space-group assignment was that originally determined from the diffraction pattern, but is perhaps complicated by translational noncrystallographic symmetry. From this aspect, it is a fascinating phenomenon that we have revealed how the CTD controls the crystallization of Pgp3. It is notable that the ordered bromine sites are in the ordered protein portions and not elsewhere (see Supplementary Fig. S8).

At this high resolution all of the amino-acid sequence differences in the CTD (namely R138S, Y191C, R210S and I212T) and some of the polypeptide-chain changes in the NTD (namely Q12E, D86N and Q90D) of this expressed form of the protein compared with that used in an earlier study were visible. The T39K and P61S changes were not visible in the electron density owing to their high *B* factors.

Our new crystal form and study adds evidence for the precise structural nature of the CTD and the NTD and for the flexibility of the linker region between them. Why there is such a complex multi-domain arrangement and the precise connection to its functionality in chlamydia currently remain unknown.

The core intrinsic macromolecular symmetry is a threefold axis which is noncrystallographic in both crystal forms. Indeed, there is not a single threefold axis but two, one through the CTD and one through the NTD, the relative orientation of which is of interest with regard to the flexible bend of the THCC.

Of special importance is the lattice interaction that is observed in both crystals involving the three Phe6 residues on the fairly flat NTD surface and one of the Trp234 residues on the curved surface of the CTD. To observe this in both crystal forms, ours and that of Galaleldeen *et al.* (2013 ▸), confirms the interest in this intermolecular interaction as being of likely functional significance in receptor binding in chlamydia. Thus, we suggest that this might allow the design of a new chemotherapeutic agent against chlamydia comprising a tryptophan-like molecule but with a higher binding affinity for these three proximal phenylalanine residues than tryptophan itself.

## Supplementary Material

PDB reference: Pgp3, 6gjt

Supplementary Figures.. DOI: 10.1107/S2052252518007637/mf5024sup1.pdf

Zip file containing structure factors and coordinates for 3 Å resolution ordered Pgp3 structure.. DOI: 10.1107/S2052252518007637/mf5024sup2.zip

Zip file containing structure factors and coordinates for 1.98 Å resolution full Pgp3 structure.. DOI: 10.1107/S2052252518007637/mf5024sup3.zip

Raw diffraction images for pgp3. URL: https://doi.org/10.5281/zenodo.1248459

## Figures and Tables

**Table 1:** Summary of the X-ray diffraction data for Pgp3 and model-restrained refinement at 1.98 Å resolution using the full 360° sample sweep data-set merge and without any NCS restraints applied Values in parentheses are for the highest resolution shell. Supplementary Fig. S7 shows the C^α^ polypeptide models for the 3 and 1.98 Å resolution cases described here and in the text. The PDB’s validation RSRZ score corroborates the disorder observed in residues 20–99. The disorder becomes a progressive effect when moving away from the interface of the CTD and NTD. Consequently, the 1.98 Å resolution refined model has a worse RSRZ score than the 3 Å resolution refined model (12.6 and 4.4%, respectively). There is no hard cutoff value for RSRZ, but we imagine that a value of around 5% is a useful guide. This led us to provide the 3 Å resolution model and also the full Pgp3 structure at 1.98 Å resolution as Supporting Information to allow consultation of the differences described. A similar situation was encountered in another study (Brink & Helliwell, 2017 ▸). This was resolved by placing a partial fully ordered structure in the PDB with a full model attached to the article. We deposited the 1.98 Å resolution refined ordered model, the details of which are in the right-hand column in the table, in the PDB.

	Data and refinement set at 1.98 Å; full Pgp3	Data and refinement set at 3 Å; ordered Pgp3	Data and refinement set at 1.98 Å; ordered Pgp3 (deposited in the PDB)
Wavelength (Å)	0.9173	0.9173	0.9173
Crystal-to-detector distance (mm)	252	252	252
Processing program	*MOSFLM*	*MOSFLM*	*MOSFLM*
PDB code			6gjt
Data-collection temperature (K)	100	100	100
Space group	*P*2_1_2_1_2_1_	*P*2_1_2_1_2_1_	*P*2_1_2_1_2_1_
Unit-cell parameters (Å, °)	*a* = 85.41, *b* = 108.25, *c* = 207.0, α = β = γ = 90	*a* = 85.41, *b* = 108.25, *c* = 207.0, α = β = γ = 90	*a* = 85.41, *b* = 108.25, *c* = 207.0, α = β = γ = 90
No. of amino acids in the whole Pgp3 trimer	792		
Molecules per asymmetric unit	2		2
Observed reflections	1536419		1536419
Unique reflections	130981	36196	130981
Resolution (Å)	45.718–1.98 (2.0–1.98)	45.718–3.0 (3.13–2.80)	45.718–1.98 (2.00–1.98)
Completeness (%)	98.1 (98.3)	97 (98)	98.1 (98.3)
*R*_merge_ (%)	20.4 (187.5)	8.7 (18.0)	20.4 (187.5)
〈*I*/σ(*I*)〉	9.7 (1.8)	55 (11.2)	9.7 (1.8)
Multiplicity	11.7 (11.0)	12 (12)	11.7 (11.0)
No. of reflections used	124215	36196	124215
*R*_free_ set	5% [6594 reflections]	5% [1954 reflections]	5% [6594 reflections]
No. of atoms			
Protein	11893	8060	8060
Bound waters	159	156	156
Br^−^	28	28	28
K^+^	6	6	6
Cruickshank DPI for coordinate error (Å)		0.4	0.16
*R* factor/*R*_free_ (%)	25.9/28.3	24.6/27.4	27.3/29.2
Ramachandran values			
Total No. of residues	1578	1034	1034
Total in core region of the Ramachandran plot	1422	995	989
Total No. of outliers	48	14	15
