# Supplementary material for: Chlamydia protein Pgp3 studied at high resolution in a new crystal form
Source: IUCrJ. 2018 Jun 7;5(Pt 4):439–48. doi: 10.1107/S2052252518007637 (PMC6038952; doi:10.1107/S2052252518007637)
Supplement: Supplementary file 1 [file m-05-00439-sup1.pdf]

IUCrJ

Volume 5 (2018)

Supporting information for article:

Chlamydia protein Pgp3 studied at high resolution in a new crystal form

Sahir Khurshid, Lata Govada, Gillian Wills, Myra O. McClure, John R. Helliwell and Naomi E. Chayen

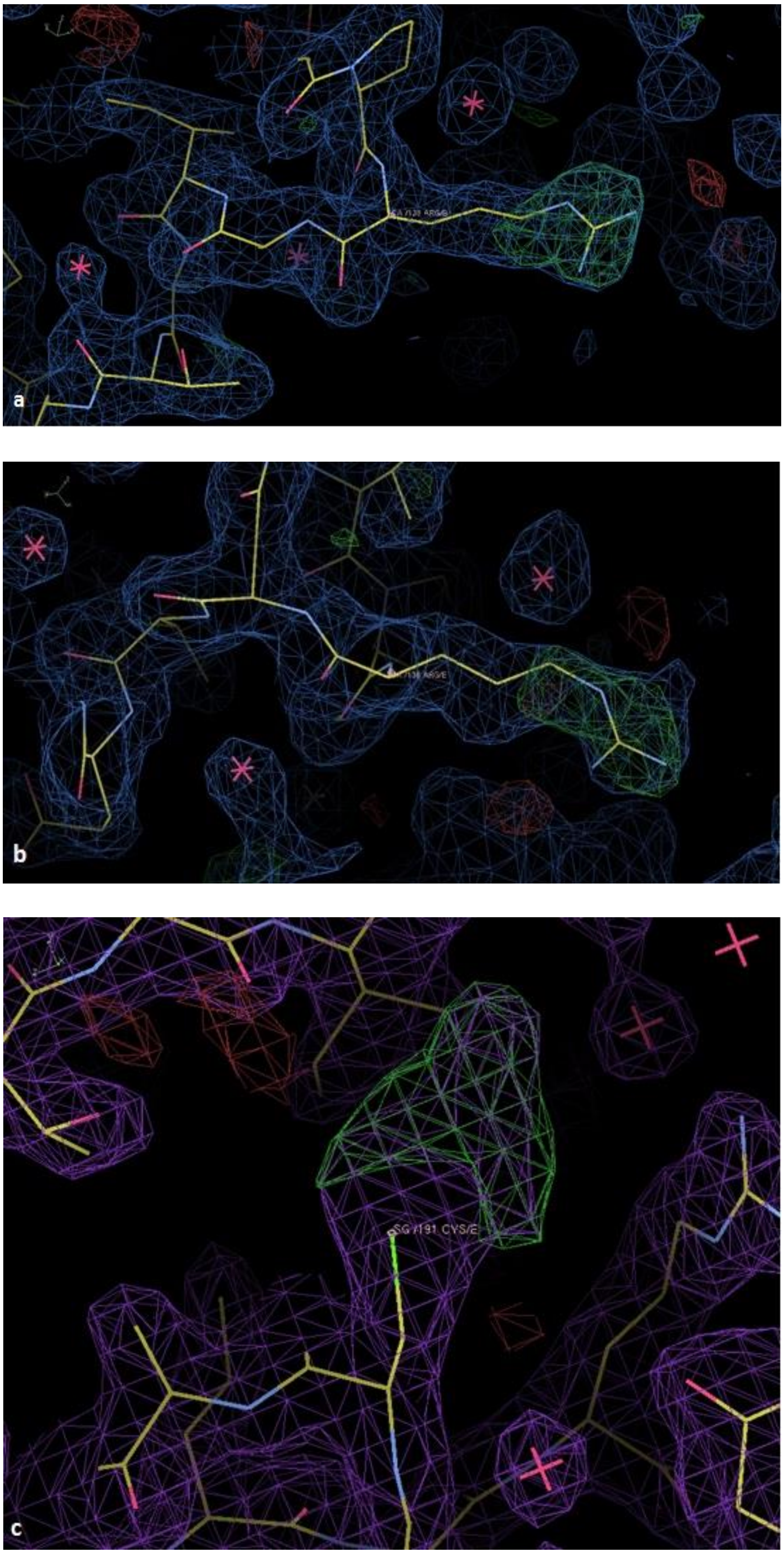

Figure S1 Example of sequence changes in Ser 138 Arg of this study versus Galaleldeen et al[15]. (a) subunit D (b) subunit E and (c) Cys191Tyr subunit E.

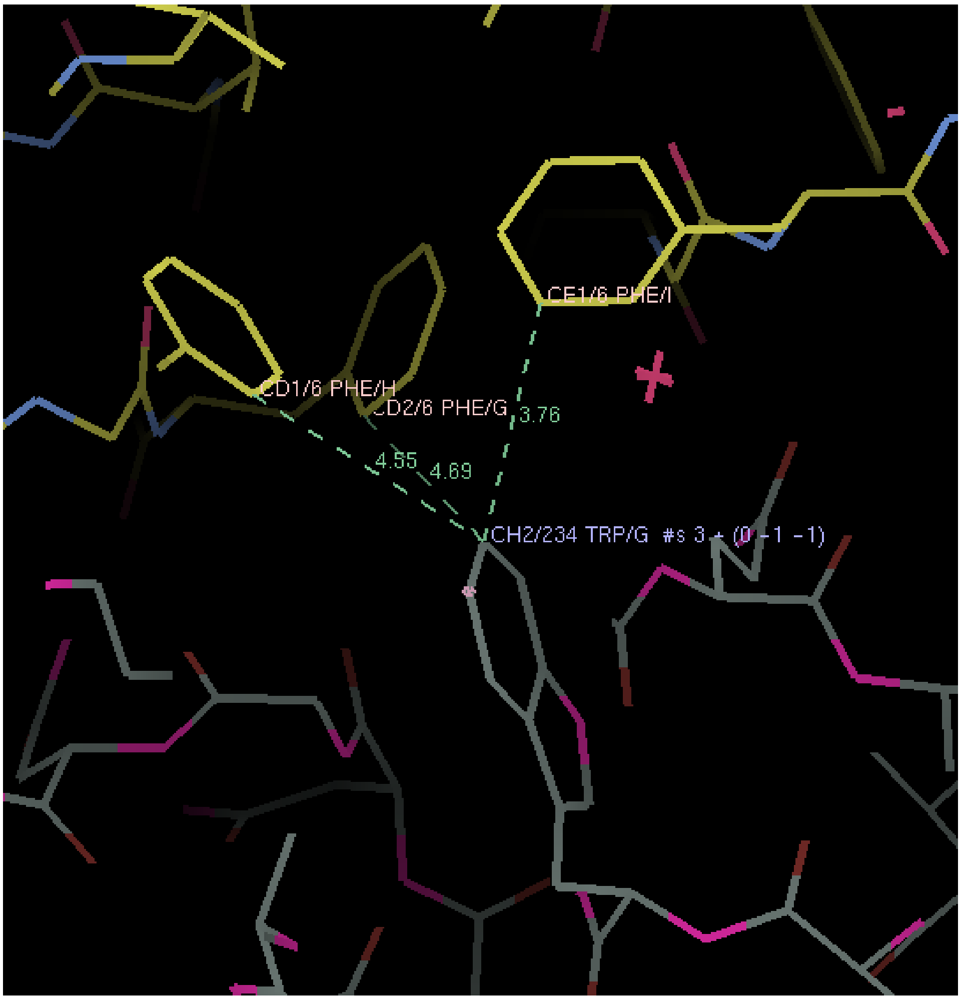

Figure S2 The Phe 6 interaction with a symmetry related A subunit Trp 234 in the 4JDO crystal structure.

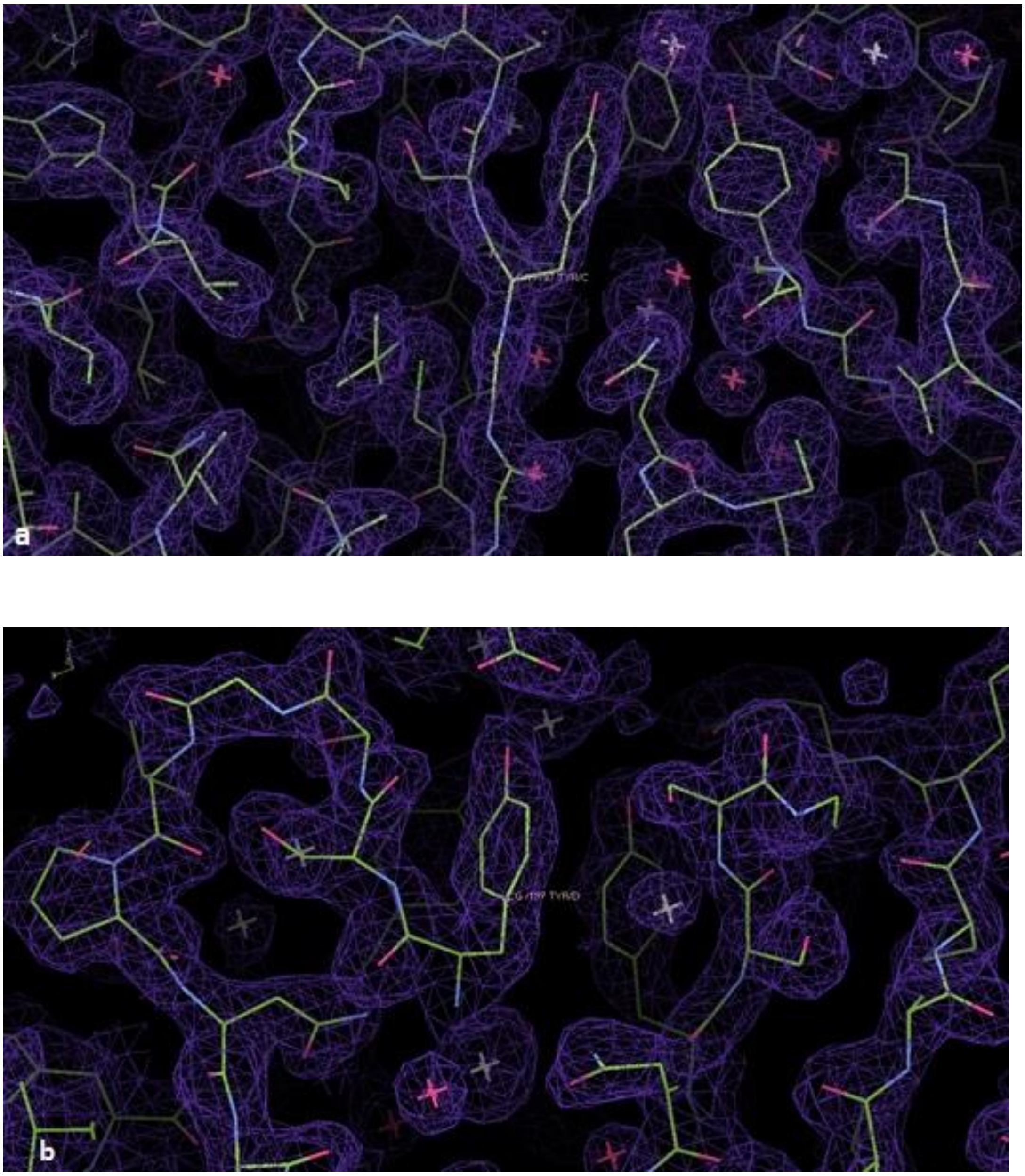

Figure S3 Composite electron density map with Tyr 197 highlighted from each Pgp3 molecule in the crystal. This figure illustrates that the CTD is well ordered. . (a) subunit C of the Pgp3 molecule 1 (b) subunit D of the Pgp3 molecule 2.

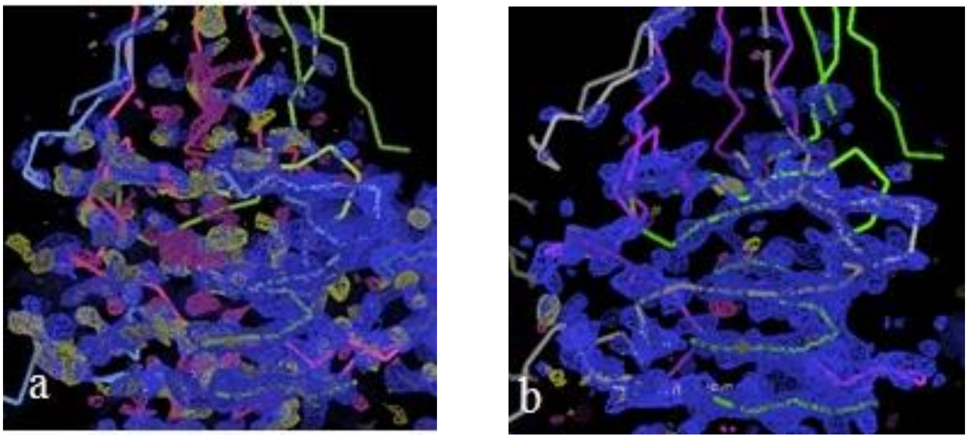

Figure S4 The NTD of the Pgp3 in molecules 1 and 2. (a) Chains A,B,C in molecule 1 and (b). Chains D,E,F in molecule 2 In yellow is the Fo-Fc electron density map (contoured from 3 sigma. In blue is the 2Fo-Fc electron density map contoured from 1.4rms.

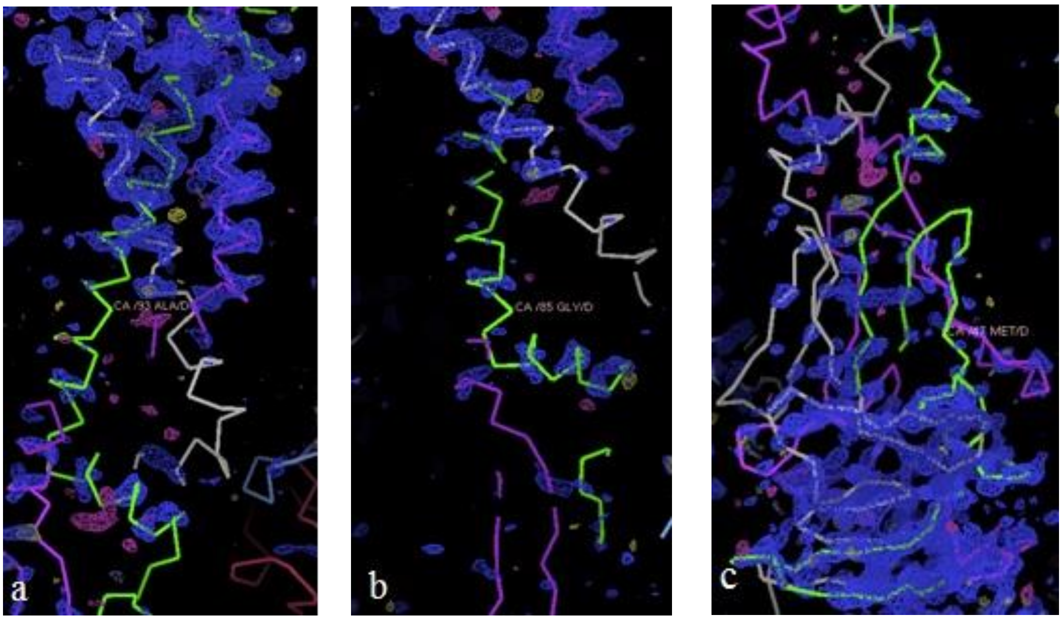

Figure S5 The THHC of one of the Pgp3 molecules. Several portions are highlighted: (a) at Ala93D (b) at Gly85 D (c) at Met47D. The orientation is with the CTD above and the NTD below. This molecule is chosen because the NTD is better ordered than the NTD in the other molecule, again as seen by the 2Fo-Fc electron density map in blue at lower portion of Fig S5c. In blue is the 2Fo-Fc electron density map contoured from 1.4rms. In yellow is the Fo-Fc electron density map contoured from 3 sigma.

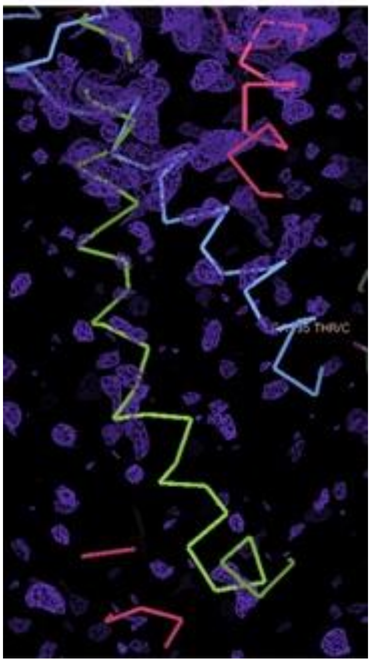

Figure S6 The THHC of the other Pgp3 molecule. One portion is highlighted namely at Thr95C to illustrate the fading out of ordered electron density as one moves from the top of the figure to the lower portion. The orientation is with the CTD above and the NTD below. In blue is the 2Fo-Fc electron density map contoured from 1.4rms. In yellow is the Fo-Fc electron density map contoured from 3 sigma.

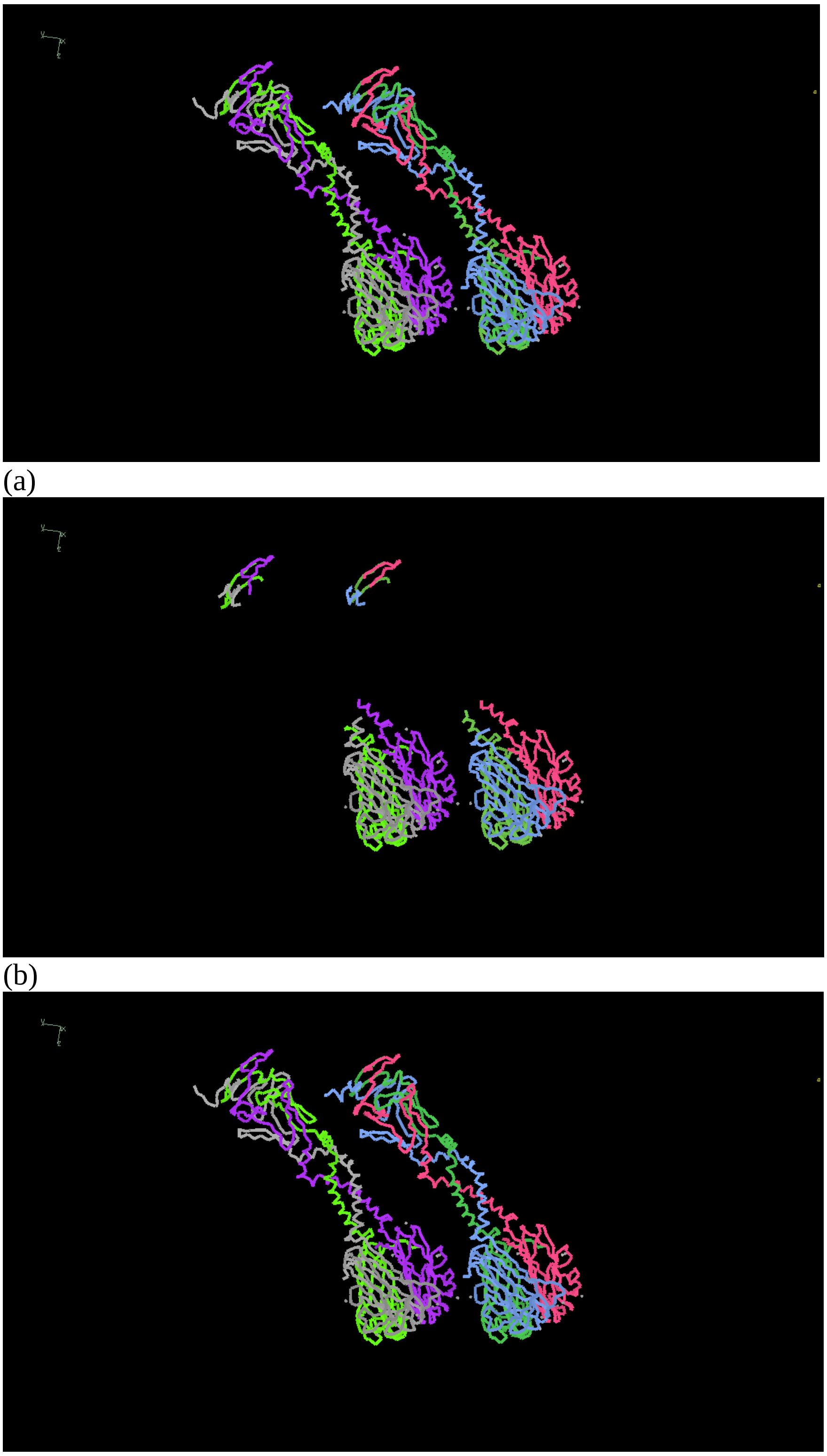

(a)

(b)

(c)

Figure S7 Cα polypeptide models for the ordered portions and the full pgp3. (a) Both models shown (b) the ordered portion of the Pgp3 and (c) the full Pgp3 model. The colour coding is to help distinguish each of the six polypeptide chains in the two Pgp3 molecules. The orientation of the Pgp3 molecules is with their CTDs at the lower part of each figure and their NTDs are at the upper part of each figure.

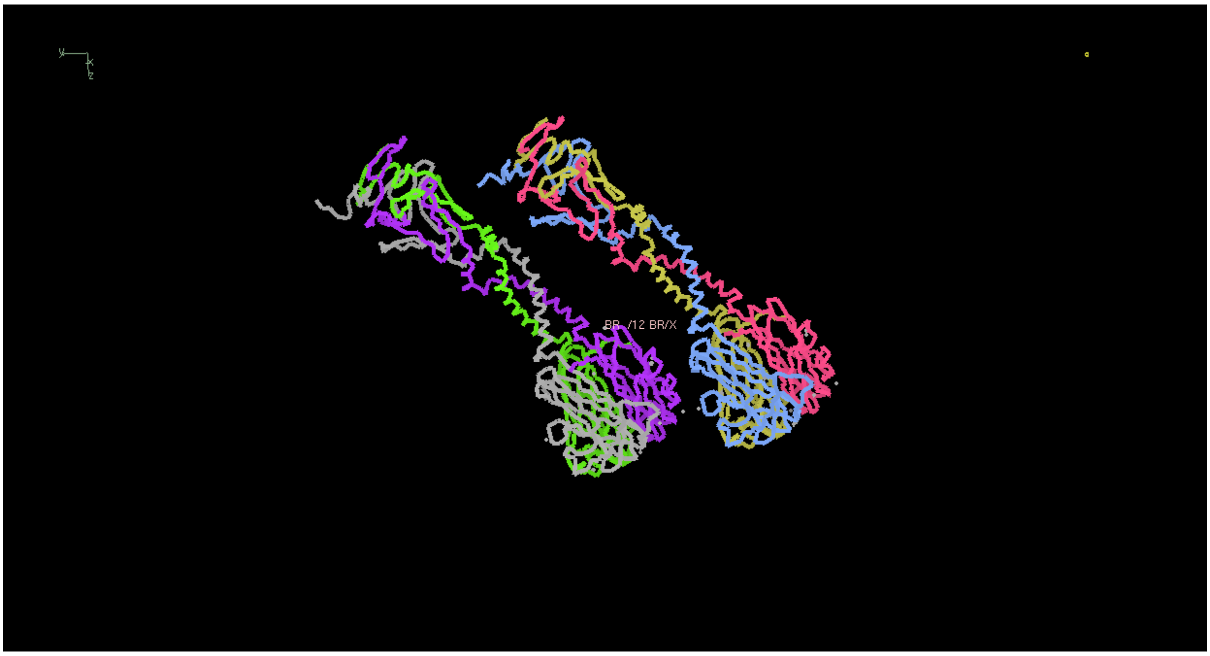

(a)

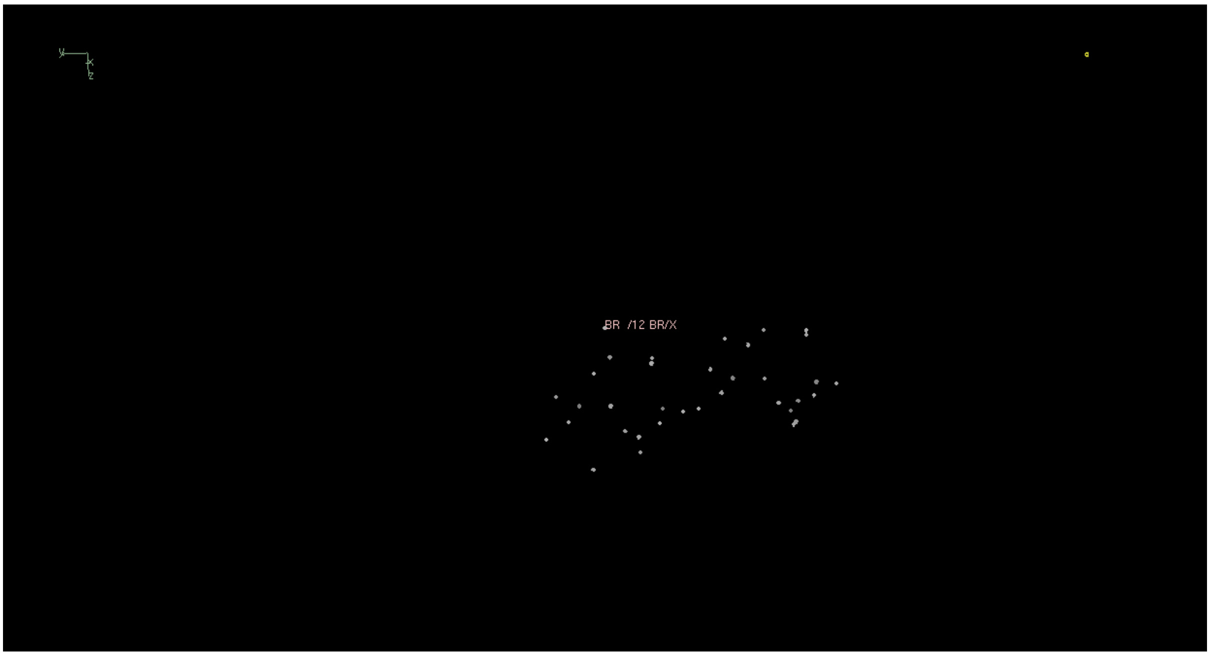

(b)

Figure S8 The anions of the pgp3 molecules. These were locatable in the CTDs, which are the most ordered portions of the Pgp3 molecules. (a) the two Pgp3 molecules in the crystallographic asymmetric unit (b) their anion sites in the same view. One of the anion sites is labelled as a reference marker.

All Supplementary figures were prepared using COOT (Emsley et al 2010).
